# Insomnia Among Caregivers of Hospitalized Patients in Medical-Surgical Wards: A Cross-Sectional Study

**DOI:** 10.7759/cureus.99596

**Published:** 2025-12-19

**Authors:** Eleni-Maria Mitrou, Niki Pavlatou, Victoria Alikari, Angeliki Stamou, Dimos Mastrogiannis, Maria Polikandrioti

**Affiliations:** 1 Department of Nursing, University of West Attica, Postgraduate Program "Applied Clinical Nursing", Athens, GRC

**Keywords:** ais, athens insomnia scale, caregivers, hospitalized patients, hospital wards, insomnia

## Abstract

Introduction: Hospitalization is a stressful event associated with physical, psychological, and financial challenges not only for patients but also for their caregivers. The aim of the present study was to explore the levels of insomnia among family caregivers of hospitalized patients in internal medicine and surgical wards and the associated factors.

Methods: In the present cross-sectional study, 160 family caregivers of hospitalized patients in internal medicine and surgical wards were enrolled. Insomnia was assessed via the “Athens Insomnia Scale” (AIS). Also, a questionnaire about demographic characteristics and caregivers’ perceptions was completed by the participants. Statistical significance was set at 0.05%.

Results: Among the 160 caregivers, 105 (65.6%) were women; 44 (27.4%) were over 60 years old; 124 (77.5%) were admitted to internal medicine wards; 47 (29.4%) had a hospitalization duration of four to six days, while 91 (56.9%) caregivers were the patients’ spouses. With respect to insomnia, the AIS recorded a mean score of 7.4 (±4.6), within a score range of 0-24, while 102 (63.7%) caregivers had an AIS score ≥6. Insomnia was associated with female gender (p=0.001), occupation (p=0.030), hospitalization of patients in surgical wards (p=0.005), staying at hospital (p=0.011), reporting extensive changes in daily routine/lifestyle, extensive illness-related financial concerns, and high uncertainty about the future (p=0.002, p=0.003, and p=0.001, respectively), as well as those who shared the room with more than two patients (p=0.001) and self-reported anxiety due to caregiving (p=0.001).

Conclusions: Healthcare professionals should be trained to assess insomnia among caregivers of hospitalized patients. Understanding caregivers’ perceptions related to their insomnia helps professionals tailor interventions, improve communication, and provide targeted education. Programs, policies, and support services work best when they are based on caregivers’ real experiences and perceptions.

## Introduction

The aging population and the increasing prevalence of chronic illnesses among older adults have intensified the role of family members as informal caregivers in healthcare provision [1]. Hospitalization, either for an acute or a chronic illness, represents a highly stressful event affecting caregivers’ well-being [1,2].

According to the model on the development of chronic sleep disorders in caregivers and care recipients proposed by McCurry, et al. the onset of insomnia and other disturbances in sleep quality among caregivers is influenced by multiple and complex factors, including physiological characteristics, life events, behavioral and environmental conditions [3]. Additionally, factors related to the care recipient, such as age, dementia status, and specific care needs, may further contribute to sleep disturbance among caregivers within the dyadic relationship. In hospital environments, the sleep of caregivers is often disturbed by factors such as noise, artificial lighting, ongoing clinical activities [1], and frequent awakenings to attend to their loved one [1,4,5].

While this disturbance is anticipated as an inevitable aspect of hospital care, insufficient sleep implies several risks and has a dual negative impact on the dyad of patient and caregiver. On the one hand, sleep deprivation significantly affects caregivers’ physical and mental health, while on the other hand, it reduces their effectiveness in providing care, thus leading to poorer outcomes for care recipients [1]. Moreover, sleep disturbances in caregivers are associated with increased stress, fatigue, impaired cognitive function, and a higher risk of developing anxiety or depression [1,2].

It is estimated that 36-50% of hospitalized patients experience sleep disturbances, which are strongly associated with increased morbidity and mortality, as well as prolonged hospital stays [1]. However, limited data exist on the sleep disturbance of caregivers during hospitalization, with the most relevant evidence focusing on caregivers of pediatric patients [5], patients with dementia [6], lung cancer [7], psychiatric disorders [8], and older adults [1]. A review showed that 50-70% of caregivers for individuals with dementia and 40% of those caring for family members with cancer experience sleep disturbances [9].

The aim of the present study was to explore insomnia among family caregivers of hospitalized patients in internal medicine and surgical wards and the associated factors (demographic, perceptions).

## Materials and methods

Study population and design

The study was conducted at the General Hospital of Athens, "Evaggelismos" in Greece. In this observational, cross-sectional study conducted from March 2025 to August 2025, 160 caregivers of hospitalized patients were enrolled. This was a convenience sample.

In this study, data were collected from participants at a single time point. Given that the study’s purpose was primarily descriptive and exploratory, focusing on identifying trends, examining associations among variables, and generating hypotheses for future research, a formal sample size calculation was not deemed necessary [10].

Insomnia is a multi-dimensional health-related issue that may aggravate or manifest within hospital settings. In the present study, caregivers’ demographics, as well as their perceptions, were included. Variables were selected to explore family caregivers’ perceptions and views, as we thought that these may influence their insomnia. Our scope was not only to identify non-modifiable factors (age, occupation, comorbidities) but also the modifiable ones (caregivers’ perceptions), which are those that provide a base for future interventions.

In the present study, family caregivers, either spouses or children, of hospitalized patients were enrolled. Understaffing and a lack of personnel in public hospitals in Greece make the presence of family caregivers essential. Additionally, in the demanding environment of the hospital, it is difficult for only one family member to stay at the bedside of the loved one [5]. Therefore, children of the family assume such responsibilities and “share the burden” with spouses/mothers. Children are younger and take on essential support roles, help with communication with health care professionals, or understand better the therapeutic regimen, as they may collect information from internet resources. On the other side, the inclusion of children as caregivers allows health professionals to understand the full support network of patients.

Inclusion and exclusion criteria

Participants were required to be able to read, write, and understand the Greek language; be capable of reading and signing the informed consent form; and have a hospitalized person in the medical and surgical wards.

The exclusion criteria of caregivers were the following: providing care to hospitalized patients diagnosed with mental disorders and cognitive impairment, and having a patient hospitalized in the intensive care unit. 

Participants were recruited exclusively from medical/surgical wards to ensure a relatively homogeneous study population. Typically, patients in these wards are considered to be more stable, having no advanced needs compared to other units. Furthermore, data collection in medical and surgical wards was more feasible, as patients were able to provide informed consent and engage in the research process.

Restricting the sample to these settings also minimized potential confounding factors associated with specialized units such as intensive care, pediatrics, or psychiatry. In these units, patient characteristics, treatment protocols, and hospital settings, including medical equipment, differ markedly.

Data collection

Data were obtained via interviews conducted during evening shifts, at times when caregivers were available and not engaged in patient care activities. Questionnaires were completed in a private office setting, with each session lasting approximately 20-30 minutes.

Research instrument

Data were collected using the Athens Insomnia Scale (AIS), which included caregivers’ characteristics. Patients’ demographic characteristics were the following: gender, age, marital status, educational level, occupation, number of children, and place of residence. With respect to patient-related characteristics, the following were recorded: the family relationship between caregiver and patient, the hospital ward to which the patient was admitted (Internal Medicine or Surgical), the duration of hospitalization, and the frequency of visits to the hospitalized patient. Further information was obtained concerning caregivers’ views. Specifically, caregivers were asked about their self-reported level of information regarding the patient’s health status, the need for written information regarding patient health, changes in daily routine, financial concerns, anxiety about caregiving, uncertainty about the future, and whether they shared a room with more than two patients.

Assessment of insomnia

The AIS was used to assess insomnia symptoms (Appendix 1). The AIS is a validated psychometric instrument designed to provide a quantitative evaluation of insomnia in accordance with the International Classification of Diseases-10 (ICD-10) diagnostic criteria [11]. It comprises eight items: the first five assess difficulties with sleep initiation, sleep maintenance, early morning awakening, and total sleep duration. The remaining three evaluate daytime functioning and well-being. Items are rated on a four-point scale (0-3), yielding a total score from 0 to 24, with higher scores reflecting greater insomnia severity. A total score of ≥6 has been proposed as the threshold indicative of insomnia. The AIS has been translated and applied across diverse populations, demonstrating strong reliability and validity, thereby supporting its use in both clinical and research settings [12,13]. The AIS has been constructed by Greek researchers in its original version with very good reliability and validity [14].

Ethical considerations

The study was approved by the Research Ethics Committee of the public hospital where the study was conducted (No. 38242/14.11.2024). Participants provided written informed consent and were informed of their right to withdraw at any time. Data were collected anonymously and confidentially in accordance with the Declaration of Helsinki (1989).

Statistical analysis

Qualitative data are presented as absolute numbers and relative (%) frequencies, while quantitative data are summarized using mean, standard deviation (SD), median, and interquartile range (IQR). The normality of distributions was assessed using the Kolmogorov-Smirnov test and graphically with histograms. The Kruskal-Wallis and Mann-Whitney tests were applied to examine associations between insomnia scores and caregivers’ characteristics. A significance level of 5% was considered statistically significant. All statistical analyses were conducted using IBM SPSS Statistics for Windows, Version 25 (released 2017; IBM Corp., Armonk, New York, United States).

## Results

Sample description

Table 1 presents the main characteristics of the family caregivers regarding the hospitalized patients they cared for. Among the 160 family caregivers, 105 (65.6%) were women; 44 (27.4%) were over 60 years old; 101 (64.4%) were married; 64 (40.0%) had a secondary education level; 51 (31.8%) were private-sector employees; 75 (46.9%) resided in the prefecture’s capital; and 54 (33.8%) had no children. Regarding patients’ characteristics, 124 (77.4%) were admitted to internal medicine wards, and 47 (29.4%) had a hospitalization duration of four to six days. In terms of kinship, 91 (56.9%) family caregivers were predominantly the patients’ spouses, while 60 (37.5%) family caregivers stayed at the hospital.

**Table 1:** Family caregivers’ characteristics (N=160)

Characteristics	n (%)
Degree of kinship	
Spouse	91 (56.9%)
Child	69 (43.1%)
Gender	
Male	55 (34.4%)
Female	105 (65.6%)
Age	
<40	36 (22.5%)
41–50	38 (23.8%)
51–60	42 (26.3%)
>60	44 (27.4%)
Marital status	
Married	101 (64.4%)
Single	37 (23.1%)
Divorced/Widowed	20 (12.5%)
Educational level	
Primary	9 (5.6%)
Secondary	64 (40.0%)
Tertiary	63 (39.4%)
MSc–PhD	24 (15.0%)
Occupation	
Household	15 (9.4%)
Public sector employee	46 (28.8%)
Private sector employee	51 (31.8%)
Self-employed	20 (12.5%)
Retired	28 (17.5%)
Place of residence	
Attica	70 (43.8%)
Prefecture capital	75 (46.9%)
Rural area	15 (9.3%)
Number of children	
0	54 (33.8%)
1	41 (25.6%)
2	43 (26.8%)
>2	22 (13.8%)
Admitted hospital ward	
Internal medicine	124 (77.4%)
Surgical	36 (22.6%)
Duration of hospitalization (Days)	
1-3	33 (20.6%)
4-6	47 (29.4%)
7-10	41 (25.6%)
>10	39 (24.4%)
Frequency of caregiver’s visits	
Staying at the hospital	60 (37.5%)
Once daily	50 (32.4%)
Twice daily	33 (20.6%)
Every other day	17 (9.5%)

Table 2 depicts family caregivers’ views regarding the care of hospitalized patients. The majority declared themselves sufficiently informed (n=77, 48.1%); 142 (88.8%) expressed the need for written information regarding the patient’s health; 80 (50.0%) reported extensive changes in their daily routine and lifestyle; 85 (53.1%) reported illness-related financial concerns (extensive and moderate); 108 (67.5%) experienced self-reported anxiety due to the caregiving role (high and moderate); 91 (56.6%) experienced uncertainty about the future after hospital discharge (high and moderate); and 110 (68.8%) shared a room with more than two patients.

**Table 2:** Family caregivers’ views regarding care of the hospitalized patients (n=160)

Views	n (%)
Level of information regarding the patient’s health status	
Very well informed	48 (30.0%)
Sufficiently informed	77 (48.1%)
Slightly informed	34 (21.3%)
Not informed at all	1 (0.6%)
Written health information needs	
Yes	142 (88.8%)
No	18 (11.3%)
Changes in daily routine and lifestyle	
Extensive	80 (50.0%)
Moderate	48 (30.0%)
Few	31 (19.4%)
None	1 (0.6%)
Illness-related financial concerns	
Extensive	45 (28.1%)
Moderate	40 (25.0%)
Few	38 (23.8%)
None	37 (23.1%)
Anxiety about caregiving	
High	53 (33.1%)
Moderate	55 (34.4%)
A little	41 (25.6%)
Not at all	11 (6.9%)
Uncertainty about the future, after hospital discharge	
High	48 (30.0%)
Moderate	43 (26.9%)
A little	40 (25.0%)
Not at all	29 (18.9%)
Shared a room with more than two patients	
Yes	110 (68.8%)
No	50 (31.2%)

Insomnia assessment

**Figure 1:**
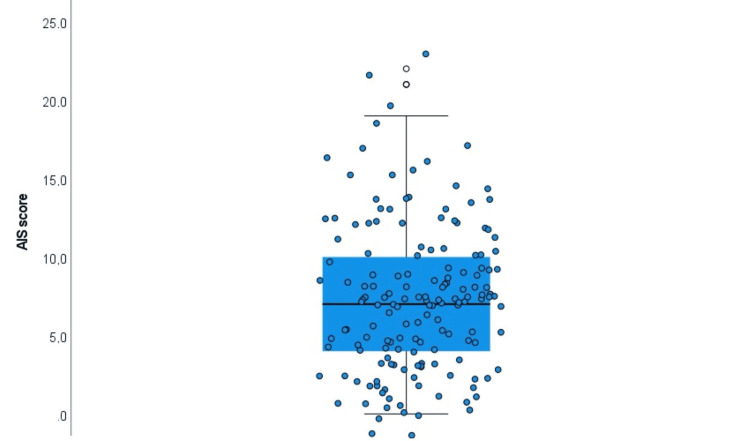
Family caregivers' insomnia measurement AIS: Athens Insomnia Scale

Table 3 and Figure 1 present results concerning family caregivers’ insomnia. The AIS (range 0-24) recorded a mean score of 7.4 (SD=4.6), with a median of 7 and an interquartile range (IQR) of 4-10. This indicates that half of the family caregivers scored below 7, and half fell within the range of 4-10. Notably, 102 (63.7%) family caregivers had an AIS score ≥6, the threshold indicative of clinically significant sleep problems. Also, the scale demonstrated satisfactory to very good internal consistency, as indicated by its Cronbach’s alpha value. Specifically, Cronbach’s alpha was 0.881 for the AIS.

**Table 3:** Description of caregivers’ insomnia (n=160) SD: standard deviation, IQR: interquartile range; AIS: Athens Insomnia Scale

Cronbach’s alpha		Mean (SD)	Media (IQR)
Insomnia (AIS) (Range 0-24)	0.881	7.4 (4.6)	7 (4-10)
Insomnia score ≥6	n (%)=102 (63.7%)		

Association of insomnia with caregivers’ characteristics

Table 4 presents the association of family caregivers’ insomnia with their characteristics. The factors found to be statistically significantly associated with insomnia were gender (AIS: p=0.001, AIS ≥6: p=0.005), occupation (AIS: p=0.030, AIS ≥6: p=0.002), hospital ward (AIS: p=0.005, AIS ≥6: p=0.005), frequency of visits (AIS: p=0.011, AIS ≥6: p=0.005), changes in routine/lifestyle (AIS: p=0.002, AIS ≥6: p=0.024), financial concerns (AIS: p=0.003, AIS ≥6: p=0.027), anxiety about the caregiving role (AIS: p=0.001, AIS ≥6: p=0.002), shared room with more than two patients (AIS: p=0.001, AIS ≥6: p=0.001), and uncertainty about the future (AIS: p=0.001, AIS ≥6: p=0.001). Regarding marital status, divorced/widowed caregivers had the lowest AIS ≥6 percentage (n=8, 7.8%) compared to married (n=70, 68.6%) and single caregivers (n=24, 23.5%).

**Table 4:** Association of insomnia with the family caregivers’ characteristics * Significance level p < 0.05%

Data	Insomnia (AIS)			AIS ≥6 (n=102)	
	Mean (SD)	Median (IQR)	p-value	n (%)	p-value
Degree of kinship					
Spouse	8.5 (4.9)	8 (5-12)	0.059	48 (47.0%)	0.183
Child	6.9 (4.4)	7 (4-9)		54 (52.9%)	
Gender					
Male	5.4 (3.7)	5.5 (2-8)	0.001*	27 (26.5%)	0.005*
Female	8.8 (4.8)	8 (5-12)		75 (73.5%)	
Age					
<40	7.4 (5.0)	7 (4-9)	0.868	28 (27.4%)	0.761
41–50	7.1 (4.6)	7 (4-9)		22 (21.5%)	
51–60	7.9 (4.4)	7.5 (5-11)		28 (27.4%)	
>60	7.9 (5.0)	7 (3-12)		24 (23.5%)	
Marital status					
Married	7.7 (4.7)	7 (5-10)	0.465	70 (68.6%)	0.029
Single	7.9 (5.0)	8 (4-11)		24 (23.5%)	
Divorced/Widowed	6.3 (4.3)	5 (3-10)		8 (7.8%)	
Educational level					
Primary–Secondary	7.9 (4.5)	7 (5-11)	0.708	48 (47.0%)	0.134
Tertiary	7.4 (4.3)	7 (5-9)		43 (42.1%)	
MSc–PhD	7.1 (6.1)	5 (2-12)		11 (10.7%)	
Occupation					
Household	6.8 (1.9)	7 (5.5-8)	0.030*	12 (11.7%)	0.002*
Public sector employee	7.5 (4.7)	7 (4-10.5)		29 (28.4%)	
Private sector employee	8.4 (4.8)	8 (5-11)		37 (36.2%)	
Self-employed	4.5 (3.5)	4 (2-7)		5 (4.9%)	
Retired	8.5 (5.4)	9 (3-12)		19 (18.6%)	
Place of residence					
Attica	8.3 (4.3)	8 (5-11)	0.131	50 (49.0%)	0.126
Prefecture capital	7.2 (5.1)	7 (4-9)		45 (44.1%)	
Rural area	5.7 (4.0)	6 (2-9)		7 (6.8%)	
Number of children					
0	7.9 (5.1)	8 (4-11)	0.365	37 (36.2%)	0.273
1	6.6 (4.5)	6 (4-8)		23 (22.5%)	
2	7.7 (4.9)	7 (4-11)		25 (24.5%)	
2	8.1 (3.6)	8 (6-11)		17 (16.6%)	
Hospital ward of the patient					
Internal medicine	7.1 (4.7)	7 (3-9)	0.005*	72 (70.5%)	0.005*
Surgical	9.3 (4.1)	9 (7-11)		30 (29.5%)	
Duration of hospitalization					
1–3 days	7.9 (5.3)	7 (3.5-11.5)	0.374	21 (20.5%)	0.096
4–6 days	6.5 (3.7)	7 (4-9)		28 (27.4%)	
7–10 days	7.7 (5.4)	7 (4-11)		22 (21.5%)	
10 days	8.4 (4.3)	8 (6-12)		31 (30.6%)	
Frequency of caregiver’s visits					
Staying at hospital	9.2 (4.8)	8 (7-12)	0.011*	48 (47.2%)	0.005*
Once daily	6.2 (4.1)	6 (3-9)		25 (24.5%)	
Twice daily	6.9 (3.8)	7 (4-9)		21 (20.5%)	
Every other day	6.9 (5.8)	6 (3-10)		8 (7.8%)	
Information about the patient’s health					
Very well informed	7.7 (5.1)	7 (4-10)	0.957	32 (31.3%)	0.419
Sufficiently informed	7.4 (4.3)	7 (4.5-9.5)		51 (50.1%)	
Slightly / Not at all informed	7.8 (5.1)	8 (3-11)		19 (18.6%)	
Written health information					
Yes	7.8 (4.7)	7 (4-11)	0.171	91 (89.3%)	0.805
No	5.9 (4.5)	7 (2-8.5)		11 (10.7%)	
Changes in daily routine/lifestyle					
Extensive	8.3 (4.5)	8 (5-12)	0.002*	57 (55.8%)	0.024*
Moderate	8.1 (4.8)	7 (5-11)		31 (30.3%)	
Few/None	4.7 (4.0)	4 (1-8)		14 (13.7%)	
Illness-related financial concerns					
Extensive	8.8 (5.2)	8 (5-12)	0.003*	30 (29.4%)	0.027*
Moderate	7.9 (4.0)	8 (5-11)		28 (27.4%)	
Few	7.9 (4.4)	8 (6-10)		28 (27.4%)	
None	5.0 (4.4)	4 (1-7)		16 (15.6%)	
Anxiety due to caregiving					
High	8.7 (4.8)	8 (5-12)	0.001*	39 (38.2%)	0.002*
Moderate	8.6 (4.2)	8 (5.5-12)		40 (39.2%)	
A little / Not at all	5.3 (4.4)	5 (2-8)		23 (22.5%)	
Shared room >2 patients					
Yes	8.7 (4.7)	8 (5.5-12)	0.001*	73 (71.6%)	0.001*
No	5.6 (4.0)	5 (3-8)		29 (28.4%)	
Uncertainty about the future					
High	9.4 (5.5)	8.5 (5-13)	0.001*	32 (31.5%)	0.001*
Moderate	8.3 (3.8)	8 (5-11)		34 (33.3%)	
A little	7.0 (4.3)	7 (3-9)		28 (27.4%)	
Not at all	4.0 (2.6)	4 (2-7)		8 (7.8%)	

Also, 102 participants had an AIS score ≥6. More in detail, children caregivers (52.9%), female (73.5%), married (68.6%), of primary and secondary education level (47.0%), private sector employees (36.2%), residents in Attica (49.0%), those having no children (36.2%), those whose patient was hospitalized in internal medicine wards (70.5%), whose patient was hospitalized > 10 days (30.6%), caregivers staying at hospital (47.2%), those who declared to be sufficiently informed (50.1%), to need written health information (89.3%), to have extensive changes in their routine (55.8%), extensive financial concerns (29.4%), moderate anxiety due to caregiving (39.2%), moderate uncertainty (33.3%), as well as those who shared a room with >2 patients (71.6%).

More specifically, women had higher levels of insomnia (median=8, IQR 5-12) compared to men (median=5.5, IQR 2-8), with 75 (73.5%) women vs. 27 (26.5%) men scoring AIS ≥6. In terms of occupation, private-sector employees and retirees exhibited higher levels of insomnia (median=8 and 9, respectively), with AIS ≥6 observed in 37 family caregivers (36.2%) and 19 family caregivers (18.6%), respectively. Conversely, self-employed family caregivers had much lower insomnia (median=4, IQR 2-7), with only five (4.9%) scoring AIS ≥6. Hospital wards also differentiated insomnia levels: family caregivers in internal medicine wards had a lower median insomnia score (7, IQR 3-9), with 72 (70.5%) having an AIS ≥6, whereas those in surgical wards had higher insomnia levels (median=9, IQR 7-11), with 30 (29.5%) having an AIS ≥6. Visit frequency was strongly associated with insomnia. Family caregivers staying at the hospital had a median score of 8 (IQR 7-12), with 48 (47.2%) having an AIS ≥6, while those visiting once daily had lower insomnia (median=6, IQR 3-9), with 25 (24.5%) having an AIS ≥6. Changes in routine/lifestyle were also related to insomnia. Family caregivers reporting extensive changes had a median score of 8 (IQR 5-12) with 57 (55.8%) having an AIS ≥6, compared to those reporting few/none, who had a lower median of 4 (IQR 1-8) with 14 (13.7%) having an AIS ≥6. Financial concerns showed a clear association with insomnia. Family caregivers without financial concerns had the lowest insomnia (median=4, IQR 1-7), with 16 (15.6%) scoring an AIS ≥6. In contrast, those reporting extensive, moderate, or few financial concerns had median scores around 8, with AIS ≥6 rates ranging from 28 (27.4%) to 30 (29.4%). Self-reported anxiety about caregiving was strongly linked to higher insomnia. Family caregivers reporting high or moderate anxiety had median scores of 8 (IQR 5-12), with 39 (38.2%) and 40 (39.2%) scoring AIS ≥6, respectively, compared to those with little/no anxiety (median=5, IQR 2-8), with 23 (22.5%) scoring AIS ≥6. Those who shared the room with more than two patients had higher insomnia (median=8, IQR 5.5-12, 73 (71.5%) scoring AIS ≥6), compared to those who did not (median=5, IQR 3-8, 29 (28.4%) scoring AIS ≥6). Finally, uncertainty about the future was strongly associated with insomnia. Family caregivers reporting high uncertainty had a median of 8.5 (IQR 5-13), with 32 (31.3%) scoring AIS ≥6. Similarly, those who reported moderate uncertainty had a median of 8 (IQR 5-11), with 34 (33.3%) scoring AIS ≥ 6. In contrast, family caregivers with no uncertainty had the lowest insomnia (median=4, IQR 2-7), with 8 (7.8%) having the lowest AIS ≥6.

**Figure 2:**
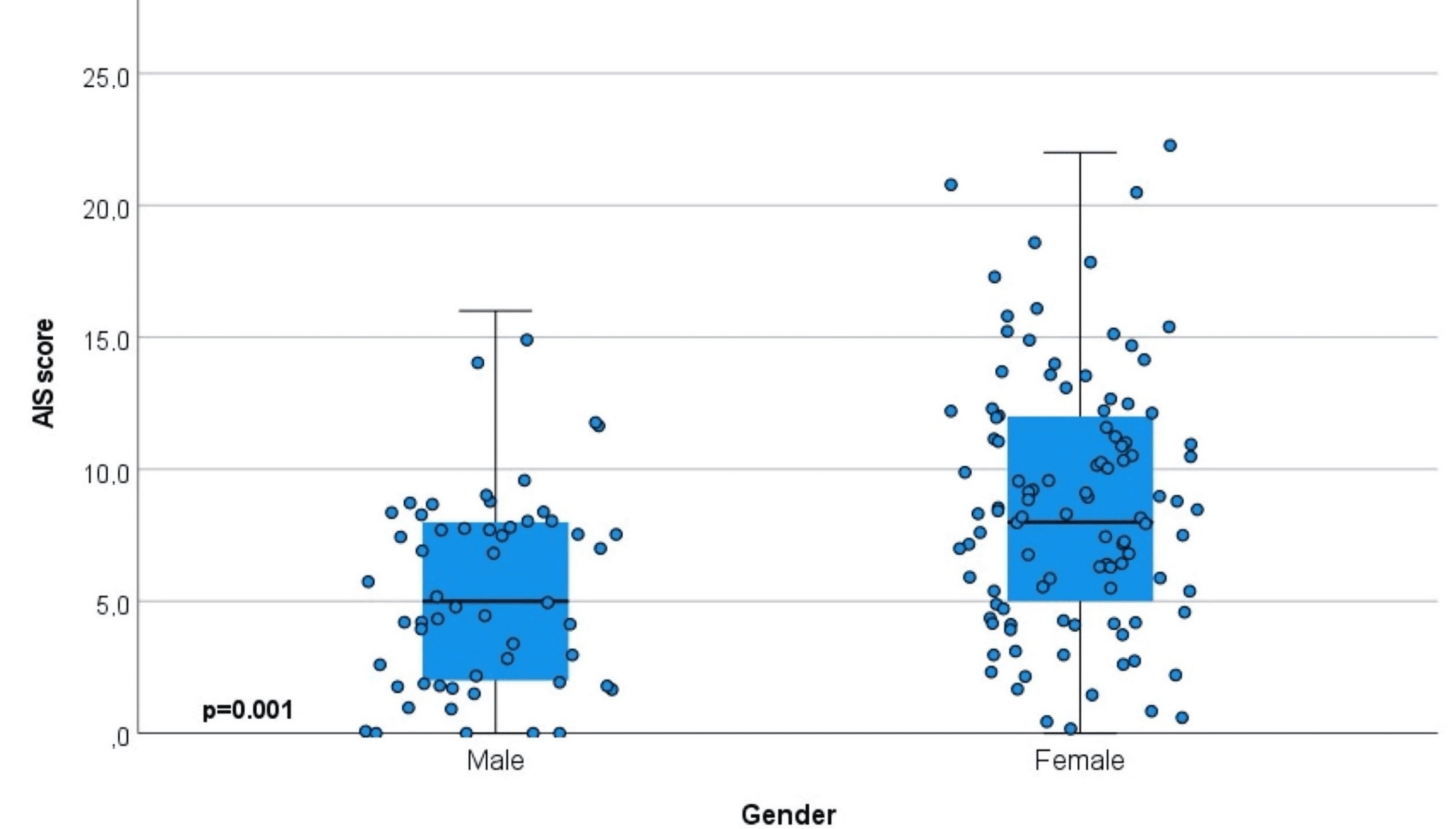
Association between insomnia and gender AIS: Athens Insomnia Scale

**Figure 3:**
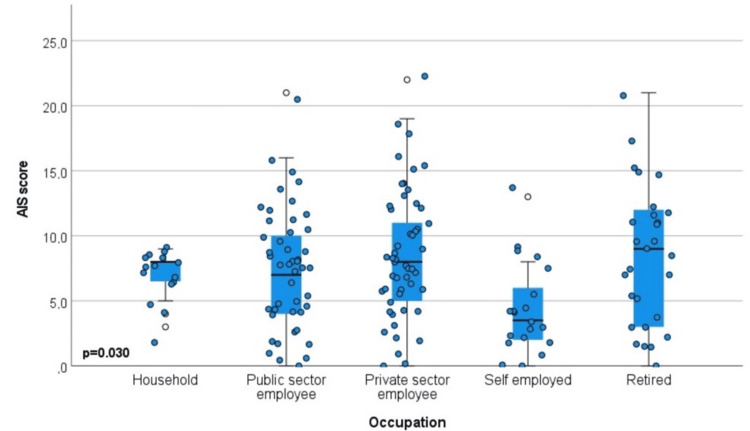
Association between insomnia and occupation AIS: Athens Insomnia Scale

**Figure 4:**
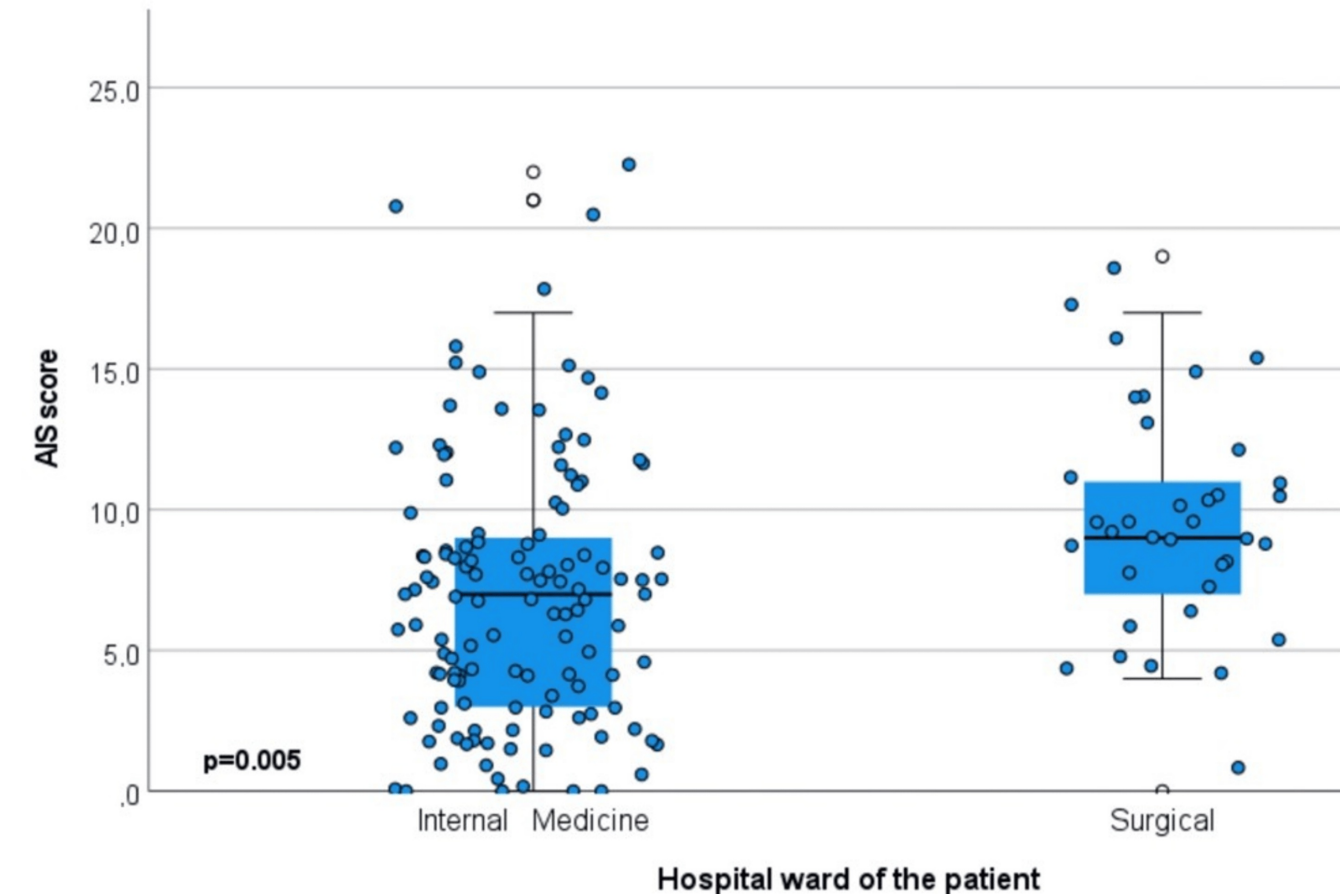
Association between insomnia and the hospital ward of the patient AIS: Athens Insomnia Scale

Box plots with jitter graphs were added to provide more insights into the distribution of the AIS score. Graphs represent three significant associations: gender (Figure 2), occupation (Figure 3), and ward of the hospitalized patient (Figure 4).

## Discussion

According to the results of the current study, caregivers experienced moderate levels of insomnia. Similarly, in Spain, Fernández-Puerta et al. demonstrated that 45.4% of informal caregivers (n=152) of hospitalized patients experienced clinical insomnia symptoms [1]. Li et al., focusing on informal caregivers of 289 hospitalized lung cancer patients, showed that 83 (28.72%), 53 (18.34%), and 14 (4.84%) experienced mild, moderate, and severe insomnia, respectively [7]. Prior studies also reported that 66% and 59% of caregivers in intensive care units and neurosurgical units, respectively, experienced sleep difficulties [15,16]. Among hospital caregivers, insomnia has emerged as a prevalent concern associated with multiple and interrelated factors. Caregivers suffering from insomnia exhibit higher caregiving burden, depression, and anxiety, as well as lower perceived social support and resilience [1,17]. According to a review by Byun et al., caregivers' characteristics, including gender, age, relationship to care recipient, and employment status, have been linked to sleep disturbances [9]. Furthermore, sleep disruption among caregivers is related to their own health status, levels of depression, anxiety, fatigue, and perceived burden. Additionally, factors related to the care recipient, including hospital transitions, dementia-related behaviors, and declining health, further contribute to caregiver sleep disturbance [9].

The present results revealed that insomnia was associated with being female, occupation (particularly private-sector employees and retirees), hospitalization in surgical wards, staying at the hospital, changes in routine/lifestyle, financial concerns, anxiety about the caregiving role, sharing the room with more than two patients, and uncertainty about the future.

Several explanations may account for the observed insomnia among female caregivers, including the interaction of biological, psychological, and social factors. Hormonal fluctuations and higher levels of stress, anxiety, and depression, as well as the strain of balancing multiple caregiving and household roles, contribute to sleep disruption. Additionally, sociocultural expectations that assign greater caregiving responsibilities to women may heighten emotional burden, further increasing the risk of insomnia [18,19]. A commonly held, yet contradictory, view is that women who have engaged in long-term caregiving at home may experience some relief from their responsibilities during hospitalization, particularly when the care recipient’s condition has recently deteriorated [1].

Although age was not associated with insomnia in the present study, research on younger and middle-aged caregivers indicates that approximately three-quarters experience poor sleep quality [9]. Similarly, the lack of association between education level and insomnia contradicts published data by Fernández-Puerta et al., who reported that caregivers with a high education level experienced five times higher levels of insomnia complaints compared to caregivers with a basic education [1].

Building upon the discussion of demographic data, self-employed participants had lower insomnia scores. This finding may reflect that the relatively limited burden of combining caregiving with professional responsibilities allows self-employed individuals to maintain more flexible routines compared to those in other occupational groups. In contrast, high workloads, long or irregular shifts, and the emotional stress of caring for patients with complex needs can disrupt normal sleep patterns [20].

Caregivers in internal medicine wards had lower levels of insomnia, whereas those in surgical wards had higher levels. A possible explanation for the increased insomnia among caregivers in surgical wards is the greater intensity and unpredictability of postoperative care. After surgery, patients often require frequent monitoring, physical assistance, and support due to reduced autonomy. Surgical procedures are also associated with an increased risk of postoperative complications, including pain, nausea, and vomiting. In contrast, caregivers in internal medicine wards typically manage more stable patients and have fewer medical tasks, resulting in lower caregiving strain and insomnia [21].

Moreover, caregivers staying at the hospital experienced insomnia. Inevitably, hospital stays involve uncomfortable sleeping conditions, irregular schedules, and frequent interruptions at night to attend to the patient’s needs, all of which can disrupt normal sleep patterns. Fernández-Puerta et al. [2] examined the association between sleep location and caregiver sleep quality during patient hospitalization [2]. Using actigraphy and sleep diaries over seven days, they found that caregivers sleeping at the hospital exhibited poorer sleep efficiency, increased sleep fragmentation, and longer periods of wakefulness after sleep onset compared to those sleeping at home.

The present descriptive analysis showed that 37.5% of caregivers were staying at the hospital. In the Greek population, a recent study showed that 45.9% of caregivers (n=340) of hospitalized patients with heart failure stayed in the hospital, which was attributed to the strong traditional family bonds, cultural influences on care provision patterns, dysfunctions of healthcare facilities, and nursing staff shortages [22]. In a relevant study, 57.2% of caregivers spent the night in the hospital with their loved ones, and most of them rested in a recliner chair at the patient’s bedside (86.6%). Sleeping at home or alternating rest between home and the hospital was associated with a lower frequency of insomnia [1].

Insomnia was also reported by caregivers who shared the room with more than two patients. In support of this view, evidence suggests that shared rooms are extrinsic factors contributing to the development of sleep problems. This effect is exacerbated when caregivers remain at the patient’s bedside overnight and when clinical activities are more frequent in shared rooms [1,2,23,24]. Patients in shared rooms in a tertiary hospital experienced the most disturbed sleep, with 51% reporting 'poor' or 'very poor' sleep quality, whereas only 17% of those in single rooms reported the same [24]. Similarly, in Australia, in 15 clinical units within a 672-bed tertiary hospital, patients reported a mean reduction of 1.8 hours in hospital sleep duration compared to home. The proportions of patients reporting their sleep quality as poor/very poor, fair, and good quality were 41.6%, 34.2%, and 24.2%, respectively [23]. Interestingly, sleep disruption extends beyond the patients themselves to caregivers, since they are also affected by environmental stressors and the demands of monitoring their loved ones. Poor patient sleep may increase caregiver nighttime activity, heightening their own risk of insomnia. This bidirectional relationship suggests that disturbed sleep environments in shared hospital settings contribute to ongoing sleep deprivation among both patients and caregivers [23,24].

A possible explanation for the finding that participants who reported changes in routine or lifestyle also reported insomnia is that disruptions to daily structure may increase vulnerability to sleep disturbances. Lifestyle disruptions, such as altered meal times, reduced physical activity, and separation from family or home responsibilities, increase psychological disturbance. These stressors contribute to hyperarousal and difficulty initiating or maintaining sleep [1].

Self-reported anxiety about the caregiving role was associated with insomnia. This emotional burden not only heightens the risk of insomnia but also exacerbates fatigue and decreases coping capacity, creating a self-perpetuating cycle of stress and sleep disturbance. A relevant study in the Greek population of relatives of hospitalized patients (n=222) demonstrated moderate levels of state and trait anxiety. Relatives of hospitalized patients experience fluctuating anxiety throughout hospitalization, influenced by childcare challenges, limited information, separation, prolonged stays, and unmet needs [25].

Of particular importance is the finding of insomnia among caregivers who reported uncertainty about the future and financial concerns, which is consistent with other relevant studies in the Greek population. Uncertainty about the future and financial concerns were associated with caregivers’ state and trait anxiety [25] as well as with quality of life [22]. Uncertainty about the future outcomes undermines coping and adaptation and often results in emotional needs being overlooked during hospitalization [25]. Worries about economic insecurity heighten anxiety and activate the body’s stress response, disrupting normal sleep regulation [22]. Unfortunately, the lack of relevant legislation in Greece effectively limits caregivers’ access to financial support from institutional services, highlighting a critical gap in policy and caregiver protection [22,26].

Including multiple variables in a study on insomnia among caregivers of hospitalized patients is crucial to capture the complexity of factors affecting sleep. Various psychological, demographic, and caregiving-related factors may influence insomnia, and assessing these variables allows for the identification of key predictors and control of confounding effects. This approach enhances the validity of the findings and supports the development of effective interventions tailored to caregivers’ needs. Therefore, the inclusion of multiple variables is critical in generating robust, clinically relevant knowledge in future studies focusing on insomnia in caregivers of hospitalized patients.

Finally, it is important to consider some confounding factors. Although hospitalization is a widely recognized source of stress, its temporary nature may prevent extreme sleep disruption. Of note are the caregiver-specific variables prior to admission, including physical health (e.g., energy levels) and emotional status (e.g., anxiety, depression), which may potentially be amplified in the clinical environment and, in turn, increase insomnia. Additionally, other factors contributing to sleep disturbances among caregivers of hospitalized patients include (a) coping strategies, such as social support, (b) guidance from hospital staff, and (c) personal habits, which include increased caffeine consumption or sleeping during the day. It is also worth emphasizing that the relationship between the caregiver and patient may impact insomnia. Finally, this research was conducted in a single general hospital in Athens, which implies that hospital-related factors may influence insomnia, such as noise and light levels, protocols including caregivers’ participation, daily routines, and clinical disruptions by health professionals.

Study limitations and strengths

This study has several limitations that should be acknowledged. First, as a cross-sectional investigation, it does not allow conclusions regarding the directionality or causality of observed associations. Second, the use of non-probability sampling limits the representativeness of the sample, restricting the generalizability of the findings to all caregivers of hospitalized patients in Greece. Additionally, data were collected from a single general public hospital, which may introduce institutional bias. Self-report measures are prone to biases such as social desirability, recall inaccuracies, and response tendencies like acquiescence and centrality, which can compromise data validity. Additionally, individuals’ subjective self-perceptions may not align with objective reality, further affecting accuracy. Expanding the study to include multiple hospitals across different regions and increasing the sample size could improve the reliability of the results and could potentially reveal additional statistically significant associations. Finally, it is important to explore caregiving responsibilities (physical, emotional, and psychological), which may significantly disrupt sleep patterns.

The strength of this study is that, to the best of our knowledge, it is the first time that levels of insomnia and its associated factors are explored among caregivers of patients hospitalized in medical or surgical wards.

## Conclusions

The present findings highlight the factors that contribute to insomnia in caregiving populations, including female gender, occupation, surgical ward, staying at the hospital, changes in routine/lifestyle, financial concerns, anxiety about the caregiving role, sharing a room with more than two patients, and uncertainty about the future.

Assessing insomnia in caregiver populations not only facilitates the identification of at-risk individuals but also informs the development of targeted interventions to improve sleep quality. Implementing self-reported monitoring of sleep through validated sleep assessment tools may provide clinicians with greater insight into sleep duration and quality. Interventions could include guidance on sleep hygiene, stress management, and strategies to enhance nighttime rest within hospital settings. Given the significant social and economic contributions of caregivers, recognizing and addressing sleep disturbances as a modifiable factor is crucial for enhancing their well-being and sustaining their caregiving capacity.

## Appendices

Appendix 1 

**Table 5:** The Athens Insomnia Scale [14]

Sleep factors	0	1	2	3
Sleep induction (the time it takes you to fall asleep after turning off the lights)	No problem	Slightly delayed	Markedly delayed	Very delayed or did not sleep at all
Awakenings during the night	No problem	Minor problem	Considerable problem	Serious problem or did not sleep at all
Final awakening	Not earlier	A little earlier	Markedly earlier	Much earlier, or did not sleep at all
Total sleep duration	Sufficient	Slightly insufficient	Markedly insufficient	Very insufficient, or did not sleep at all
Sleep quality	Satisfactory	Slightly unsatisfactory	Markedly unsatisfactory	Very unsatisfactory, or did not sleep at all
Well-being during the day	Normal	Slightly decreased	Markedly decreased	Very decreased
Functioning capacity during the day	Normal	Slightly decreased	Markedly decreased	Very decreased
Sleepiness during the day	None	Mild	Considerable	Intense
